# Feature engineering for sentiment analysis in e-health forums

**DOI:** 10.1371/journal.pone.0207996

**Published:** 2018-11-29

**Authors:** Jorge Carrillo-de-Albornoz, Javier Rodríguez Vidal, Laura Plaza

**Affiliations:** UNED IR & NLP Group, Madrid, Spain; Arizona State University, UNITED STATES

## Abstract

**Introduction:**

Exploiting information in health-related social media services is of great interest for patients, researchers and medical companies. The challenge is, however, to provide easy, quick and relevant access to the vast amount of information that is available. One step towards facilitating information access to online health data is *opinion mining*. Even though the classification of patient opinions into positive and negative has been previously tackled, most works make use of machine learning methods and bags of words. Our first contribution is an extensive evaluation of different features, including lexical, syntactic, semantic, network-based, sentiment-based and word embeddings features to represent patient-authored texts for polarity classification. The second contribution of this work is the study of *polar facts* (i.e. objective information with polar connotations). Traditionally, the presence of polar facts has been neglected and research in polarity classification has been bounded to opinionated texts. We demonstrate the existence and importance of polar facts for the polarity classification of health information.

**Material and methods:**

We annotate a set of more than 3500 posts to online health forums of breast cancer, crohn and different allergies, respectively. Each sentence in a post is manually labeled as “experience”, “fact” or “opinion”, and as “positive”, “negative” and “neutral”. Using this data, we train different machine learning algorithms and compare traditional bags of words representations with word embeddings in combination with lexical, syntactic, semantic, network-based and emotional properties of texts to automatically classify patient-authored contents into positive, negative and neutral. Beside, we experiment with a combination of textual and semantic representations by generating concept embeddings using the UMLS Metathesaurus.

**Results:**

We reach two main results: first, we find that it is possible to predict polarity of patient-authored contents with a very high accuracy (≈ 70 percent) using word embeddings, and that this considerably outperforms more traditional representations like bags of words; and second, when dealing with medical information, negative and positive facts (i.e. objective information) are nearly as frequent as negative and positive opinions and experiences (i.e. subjective information), and their importance for polarity classification is crucial.

## Introduction

Patients, and citizens in general, are increasingly using the Internet for searching health information and support. Eurobarometer reports that six out of ten European citizens looked for health-related information online in 2014, 92% of whom reported that they would continue using the Internet as a primary source of health-related information in the future [1]. According to Google, one in 20 searches is for health information (Google blog: http://googleblog.blogspot.co.uk/2015/02/health-info-knowledge-graph.html). Fox and Jones [2] also reported that 80% of Internet users have searched for health topics, such as specific symptoms or treatments, 34% of them have read someone else’s commentary or experience on blogs, social networks and health communities, and 24% of them have consulted online reviews of particular drugs or treatments.

The use of online health communities is particularly popular among chronic patients. Surveys show that these patients significantly benefit from social interaction with peers and the sharing of knowledge, experiences and support [3, 4]. Evaluations of peer-led self-management programs using social media for several chronic diseases indicate positive outcomes and promise to complement the provision in the given health system [5–7].

Information in online health forums and communities is also of great interest for researchers and professionals, as it allows for investigations in naturalistic settings, which cannot easily be replicated in laboratory environments. Secondary effects of drugs, alternative therapies, undocumented symptoms, social alarms, are just some examples. The health industry is also an important stakeholder. Pharmaceutical companies, for instance, mine online health information to monitor patients’ opinions on their products and services, and to obtain feedback on their performance and the consumers’ satisfaction.

However, the amount of information is so vast that it is difficult for the users to find the information that they need. One step towards easier access to relevant information is *opinion mining*, that is frequently understood as classifying a fragment of text (phrase, sentence, or document) in polarity classes such as positive, neutral or negative. To address this problem, several unsupervised and supervised methods have been proposed. Traditionally, the supervised methods have achieved significantly better results and have been implemented using machine learning techniques, the main challenge being the identification of the appropriate signals or features for the algorithms. However, to the best of our knowledge, no previous work have evaluated the effectiveness of novel approaches based on word embeddings in order to extract features from e-Health forums. To this end, our aim is to evaluate to what extent word embeddings which have been widely used in other classification tasks and domains, may be applied to patient-authored content, along with other traditional lexical, grammatical, semantic, network-based and sentiment-based features. To do this, we evaluate its effectiveness to a very well-known task known as polarity classification; in particular, to the classification of sentences from online health forums into positive, negative and neutral.

The second contribution of this work is to do with the study and classification of factual information with polar orientation. There are a lot of state-of-the-art social media analytic tools for extracting users’ opinions [8–12]. A few recent works have also dealt with the classification of patient-authored content in polarity classes (negative vs. positive) [8, 13, 14]. However, these works assume that this classification is only applicable to subjective texts (i.e., to “opinionated information”) but not to objective texts (i.e., to “factual information”). From our point of view, this separation is not correct: as other previous works have noted [15, 16], in certain application domains facts may also have polar orientations, since they may have negative/positive implications for users (patients, in our case). In this way, for instance, the sentence *“Taking Lialda makes people vomit very often”* describes a fact with obvious negative implications for patients, while the sentence *“If the cancer is located only in the breast, the 5-year relative survival rate of people with breast cancer is 99%”* describes a clearly positive fact. We will refer to these facts with polar orientation as **polar facts**. Our intuition is that such polar facts are particularly frequent when dealing with medical information, and that they play an important role, as in other application domains, in the extraction of relevant information. Therefore, we want to test how easy it is to classify such facts in polarity classes in health-related text and to what extent they enclose relevant information.

In summary, our work presents some novel and valuable contributions toward the main objective of making online health information accessible to all the stakeholders:

- First, we present a detailed study comparing traditional bags of words with word embeddings, and their combination with other lexical, semantic, network-based and sentiment-based features. In addition, we also propose the use of domain-specific concept embeddings from the UMLS Metathesaurus and evaluate its efficiency. To the best of our knowledge, no previous work have used domain concept embeddings for the automatic classification of patient-authored contents.
- Second, we present a manually annotated dataset of online discussions concerning three different diseases: allergies, crohn and breast cancer. Our annotation compromises polarity (positive, negative and neutral) as well as factuality (opinions, facts and experiences).
- Third, we demonstrate the existence and importance of polar facts for the polarity classification of health information. To this end, we present a study on the frequency of negative/positive/neutral statements in conversations from online health forums and their classification into three factuality classes: facts, opinions and experiences. Our study demonstrates that negative and positive facts (i.e. objective information) are nearly as frequent as negative and positive opinions and experiences (i.e. subjective information).

The rest of the article is organized as follows. First, we present some related work in the area. Second, we describe the *eDiseases dataset*, the machine learning methods and the classification features used in our experiments. Third, we present the evaluation framework and results. Fourth, we discuss the obtained results. Finally, we draw the main conclusions of the study and outline future work.

## Related work

### Sentiment analysis and polarity classification

Sentiment analysis is the research area that analyzes people’s opinions, sentiments, evaluations, appraisals, attitudes, and emotions towards entities [17].

Sentiment analysis has traditionally involved two main tasks: subjectivity analysis and polarity classification. Subjective analysis refers to the classification of a given text (usually a sentence) into one of two classes: objective or subjective, i.e., separating facts from feelings, views, or beliefs. Polarity classification faces the problem of determining whether a text entails positive or negative connotations.

Sentiment analysis has been extensively applied to the automatic classification of online product reviews. The majority of existing approaches employ machine learning algorithms (particularly Naive Bayes, support vector machines and regression models) to build classifiers that assign a polarity class to each review (or sentence within the review) [18–21]. The focus of most works is the selection of the appropriate features to improve the classification performance.

The most widely used classification feature is the bag of words, computed as the TF-IDF values of words [19, 22]. Also very frequent is the use of sentiment-based lexicons to detect positive, negative and neutral words within the text, as well as subjective words and emotions, whose frequencies are later used as classification features [23–25]. Some popular lexicons are the General Inquirer [26], SentiSense [27] and SentiStrength [28].

The use of grammatical features, such as the part of speech of words and the number of adjectives, nouns, verbs and adverbs is also quite common [29, 30]. Other works have addressed the presence of negation (e.g., not good) and intensification (e.g., very good) [31, 32], often referred to as contextual valence shifters.

More sophisticated are the approaches that exploit the textual structure of the documents [33, 34]. As stated by [35], structural aspects may contain valuable information. For instance, sentiment-carrying words in a conclusion may contribute more to a text’s overall sentiment than sentiment-carrying words in, e.g., background information.

Several works have proposed the use of Latent Semantic Analysis (LSA) in different sentiment analysis tasks. LSA uses singular value decomposition (SVD) to decompose a large term-document matrix into a set of k orthogonal factors, thus transforming the original textual data to a smaller semantic space by taking advantage of some of the implicit higher-order structure in associations of words with text objects [36].

More recently, deep learning models have achieved remarkable results in different NLP tasks [37], including sentiment analysis. [38] proposes a convolutional neural network model with one layer of convolution on top of word vectors obtained from an unsupervised neural language model (words embeddings by [39]). The model is applied to prediction of positive/negative customer reviews of various products. [40] propose a multichannel and variable-size convolution neural network (CNN) architecture for sentence classification, that combines diverse versions of pre-trained word embeddings. The network is applied to two tasks with promising results: Twitter sentiment prediction and subjectivity classification. [41] introduce a models that first learns sentence representation with convolutional neural network, and after, semantics of sentences and their relations are adaptively encoded in document representation with gated recurrent neural network. The model is applied to document level sentiment classification, outperforming state-of-the-art methods. Other works have also used word embeddings to classify sentiments in tweets [42] and online product reviews [43, 44], with promising preliminary results.

### Sentiment analysis and polarity classification in patient-authored texts

Very little research has been done in sentiment analysis on health-related forums. Several previous works have tried to classify patient-authored content into positive, negative and neutral [8, 13, 14]. However, most works assume that this classification is only applicable to subjective texts (i.e., to “opinionated information” but not to “factual information”). As already mentioned, from our point of view, this separation is not correct. In certain application domains, facts may also have polar orientations, since they have negative implications for users. Moreover, these polar facts may be quite frequent and may contain very relevant information for the final users.

In [8], subjective sentences from online discussions about hearing aids are classified into positive, negative and neutral, using traditional sentiment analysis features such as the number of subjective words in the sentence, the number of adjectives, adverbs and pronouns, and the number of positive, negative and neutral words, as found in the Subjectivity Lexicon [45]. These features are used to train and test a Naive Bayes, a SVM and a logistic regression model. The best result was obtained using logistic regression (0.68 F-1).

In [13], a medical lexicon is presented that contains user reviews on drugs and medications labeled with a polarity value from 0 to 10 (0-very negative, 10-very positive). This lexicon includes both opinion words from the general domain and medical-specific opinion words along with their polarity. The general-domain lexicon is built by merging and improving existing lexicons such as the Subjectivity Lexicon and SentiWordNet, while the medical opinion lexicon is built using a corpus of drug reviews manually labeled with a polarity value. The lexicon is used to perform polarity classification on subjective contents (opinions), achieving a F-1 of 0.62 for the positive class, 0.48 for the negative class and 0.09 for the neutral class.

In [14], Twitter messages are analyzed to label subjective health information as positive, negative or neutral. To this end, the authors trained different Naive Bayes, Decision trees, KNN and SVM algorithms on different bags of words configurations. Their best configuration achieved up to 69% F-1. Again, polarity classification is restricted to subjective information, disregarding polar facts, which are very frequent and relevant in our application domain.

In [46], the aim is to separate informative from affective information in medical web-logs, medical reviews and Wikis. To this end, the authors present a method to classify blogs based on their information content. The work exploits high-level features, such as concepts and semantic types from the Unified Medical Language System (UMLS) as well as simple bags of words. They get a F-1 of up to 77% in the differentiation of affective versus informative posts.

### Words embeddings for textual representation

Vector-based models for word representation arise to overcome the limitation of traditional representations based on indices in a vocabulary. These representations fail to capture relations and similarities between words. In contrast, vector-based models may encode continuous similarities between words as the distance between word vectors in a high-dimensional space.

Vector-based models have proven useful in tasks such as word sense disambiguation, named entity, recognition, part of speech tagging, document retrieval, and sentiment analysis [37, 43, 47, 48].

A particular form of vector-based model that is receiving increasing attention by the NLP community is the word embeddings model. Word embeddings have been used to reduce data sparsity in the training data for supervised learning, achieving a significant increase in accuracy. Each dimension of the word vector represents a feature of the word, that usually has a semantic and/or syntactic interpretation. Word embeddings are typically induced using neural networks [37, 39, 49, 50].

Word embeddings have been also explored for health-related information analysis. Jagannatha and Yu [51], for instance, use skip-gram word embeddings to initialize the input layer of a recurrent neural network (RRN), and also as a feature to a conditional random field model. The embeddings are trained on a large unlabeled biomedical dataset, compiled from three sources, the English Wikipedia, an unlabeled EHR corpus, and PubMed Open Access articles. They apply both models to the detection of medical events (including medication, diagnosis, and adverse drug events). Results show that RNN significantly out-performed the CRF models.

Zou et al. [52] use a Twitter data set to train word embeddings and use them as features to both a regularised linear (Elastic Net) and a nonlinear (Gaussian Process) regression function for the surveillance of infectious intestinal diseases in social media. Nikfarjam et al. [53] learn word embeddings from more than one million unlabeled user posts from DS and Twitter, and use them as a feature to a conditional random field, along with other lexical and semantic features, for pharmacovigilance. Dubois and Romano [54] use a combination of natural language processing and deep learning techniques to develop models that can learn embeddings of clinical terms and notes, that can be later used in multiple applications.

DoctorAI [55, 56] use a recurrent neural network trained on electronic health records to predict future occurrences of diseases, and [57] employs a recurrent model to extract phenotypes from medical data, regularizing it with prior medical knowledge.

In [58], prescriptions from discharge summaries are extracted using word embeddings and conditional random fields, while De Vine et al. [59] study the application to clinical concept extraction of a specific unsupervised machine learning method, called the Skip-gram Neural Language Model, combined with a lexical string encoding approach and sequence features.

## Material and methods

### The eDiseases dataset

The eDiseases dataset (https://zenodo.org/record/1479354) contains patient-authored data from the MedHelp health site, which comprises more than 170 communities devoted to different diseases or health conditions (such as diabetes, pregnancy, obesity, cancer, etc.). Each community consists of a number of conversations; a conversation being a sequence of comments posted by patients.

To build the dataset, we automatically extracted 10 conversations from each of the following three communities: allergies, crohn and breast cancer. We selected a set of diseases that, according to medical expert, show high heterogeneity concerning both the degree of medical understanding of the diseases and the profile of the users:

- **Allergic diseases** include a number of hypersensitivity conditions whose causes are not clearly determined, the symptoms are very different and unspecific, the reactions may vary from very mild to life-threatening, diagnosis is difficult and the treatments and prevention mechanisms still generate some medical controversy. Patients are both men and women of any age.
- **Crohn’s disease** is a chronic condition that may limitate the daily life of the patients and make people feel stressed and depressed. Although symptoms are well defined, it may be very similar to other conditions such as ulcerative colitis. Since there is no cure for this disease and treatments are not always effective, alternative therapies are very common. Crohn’s is more prevalent among adolescents and young adults between the ages of 15 and 35.
- **Breast cancer** is a more well understood disease, where diagnosis and treatments are highly standardized, and the symptoms are usually the same (the presence of a lump that feels different from the rest of the breast tissue) although other more complex symptoms may be present. Patients are mostly women and usually over the age of 40.

The conversations were selected randomly, but we automatically filtered out conversations with less than 10 posts. In total, we extracted 146 posts for allergies, 191 posts for crohn, and 142 posts for breast cancer; which include 983 sentences for allergies, 1780 sentences for crohn, and 1029 sentences for breast cancer, covering a 6 years time interval.

Three frequent users of health forums annotated each sentence in the dataset as:

- “Opinion”, “Fact” or “Experience”, and
- “Positive”, “Negative” or “Neutral”.

Clear instructions were dictated by a domain expert to the three annotators that were guided through a training process. Doubts were consulted and discussed all through the annotation process. Examples were given to clarify the distinction among the different categories:

- A **fact** is something that can be checked and backed up with evidence. A fact can be verified. Examples of factual sentences from the dataset are *“Most of our gloves and supplies are latex free, now”* and *“An upper endoscopy (to look at the esophagus, stomach and small intestine) is another test to rule out Crohn’s as biopsies can be taken of the tissue during the procedure”*. However, it is important to point out that, since users in the networks are not medical experts, some of the “facts” stated by them may not be completely true. When labeling the dataset, we do not accomplish any verification process.
- An **opinion** is a judgment, viewpoint, or statement that is not conclusive. An opinion is not always true and cannot be always proven. Examples of opinionated sentences are *“I think you should see an allergist for some skin and RAST tests to help identify your allergy and any other unknown possibilities, too”* and *“It is not an IBD auto-immune disease and in my opinion, a lazy diagnosis by doctors who cannot be bothered to do proper evaluations”*.
- An **experience** is something someone has lived through and that leaves an impression on her. It is expected to be true (and in this sense is near to the concept of fact), but may be affected by personal impressions and sentiments (so it may include subjective appraisals as in the case of opinions). Examples of sentences describing experiences are *“I was diagnosed with it after my blood work, colonoscopy and biopsies came back positive and after living a nightmare”* and *“I had to take prescription strength Benadryl this morning because of some delayed reaction to something that was making it impossible to sleep because of the reflux”*. We have added this new category to the traditional categorization of facts *vs*. opinions because our manual inspections on chat room discussions about illnesses show that experiences are widely shared among the community members (even more than opinions). Instead, when looking for medical facts patients usually visit contrasted websites such as MedlinePlus.

It is possible that, when describing an experience, the user also expresses an opinion, so that experiences are not always mutually exclusive from opinions. If an experience includes an opinion, the annotator is asked to label the sentence as “experience”.

On the other hand, “facts”, “opinions” and “experiences” may be **positive**, **negative** or **neutral**, depending on whether they express or arose positive, negative or neutral sentiments and feelings, respectively. Table 1 shows examples of positive, negative and neutral facts, opinions and experiences.

**Table 1:** Examples of sentences from the *eDiseases* dataset according to their factuality and polarity.

**Facts**	
***Positive***	*Mesalamine, Lialda, Asacol etc., do not correlate with hair loss*.
***Negative***	*Recent studies link prednisone to hip degeneration, osteoporosis, to name a few*.
***Neutral***	*An upper endoscopy (to look at the oesophagus, stomach and small intestine) is another test to rule out Crohn’s*.
**Experiences**	
***Positive***	*My colonoscopy and endoscopy came back negative for Crohn’s*.
***Negative***	*I went in to food allergists that literally wouldn’t test me for allergies because they didn’t think I had enough “evidence” that I was allergic*.
***Neutral***	*Hi I went for my second opinion, not sure what to make of the appointment though*.
**Opinions**	
***Positive***	*I am glad you posted about rebound reactions because yes sure enough my welts have returned, not as bad as they first were*.
***Negative***	*I know how scary it is, and how alone you can feel*.
***Neutral***	*The best advice I could give is to watch what you eat, make a good diary of what you eat and when and how it makes you feel, also include how it affects your bowel movements*.

In case of doubt, we asked the annotators not to label the sentences. To choose one label for each sentence and category, we adopted the following guidelines:

1. If at least two annotators assigned the same label to the sentence, then such label was finally assigned.
2. If each of the three annotators assigned a different label to a sentence, then a fourth annotator was asked to select the final label.
3. If a sentence was not labeled by at least two annotators, we preserve the sentence for readiness and labeled it as NOT_LABELED.

As a result, we collected 967 labeled sentences for allergies, 1,709 labeled sentences for crohn, and 959 labeled sentences for breast cancer.

Tables 2 and 3 show the distribution of sentences into factuality and polarity classes. It may be observed from Table 2 that most sentences are experiences, followed by opinions and, finally, facts. This was expected, given that they come from communities of patients that mainly share their experiences in the management of their health condition. When searching for facts about an illness, instead, patients usually visit web pages from accredited medical institutions. On the other hand, Table 3 shows that most sentences are neutral, followed by negative sentences and, finally, positive sentences.

**Table 2:** Distribution of sentences into information types (“Facts”, “Experiences” and “Opinions”).

	Facts	Experiences	Opinions
**Allergies**	267	348	271
**Crohn**	273	931	389
**Breast cancer**	225	278	310
**Total**	765	1,557	970

**Table 3:** Distribution of sentences into polarity classes (“Positive”, “Negative” and “Neutral”).

	Positive	Negative	Neutral
**Allergies**	162	294	499
**Crohn**	302	614	712
**Breast cancer**	171	216	475
**Total**	635	1,079	1,686

**Fig 1:**
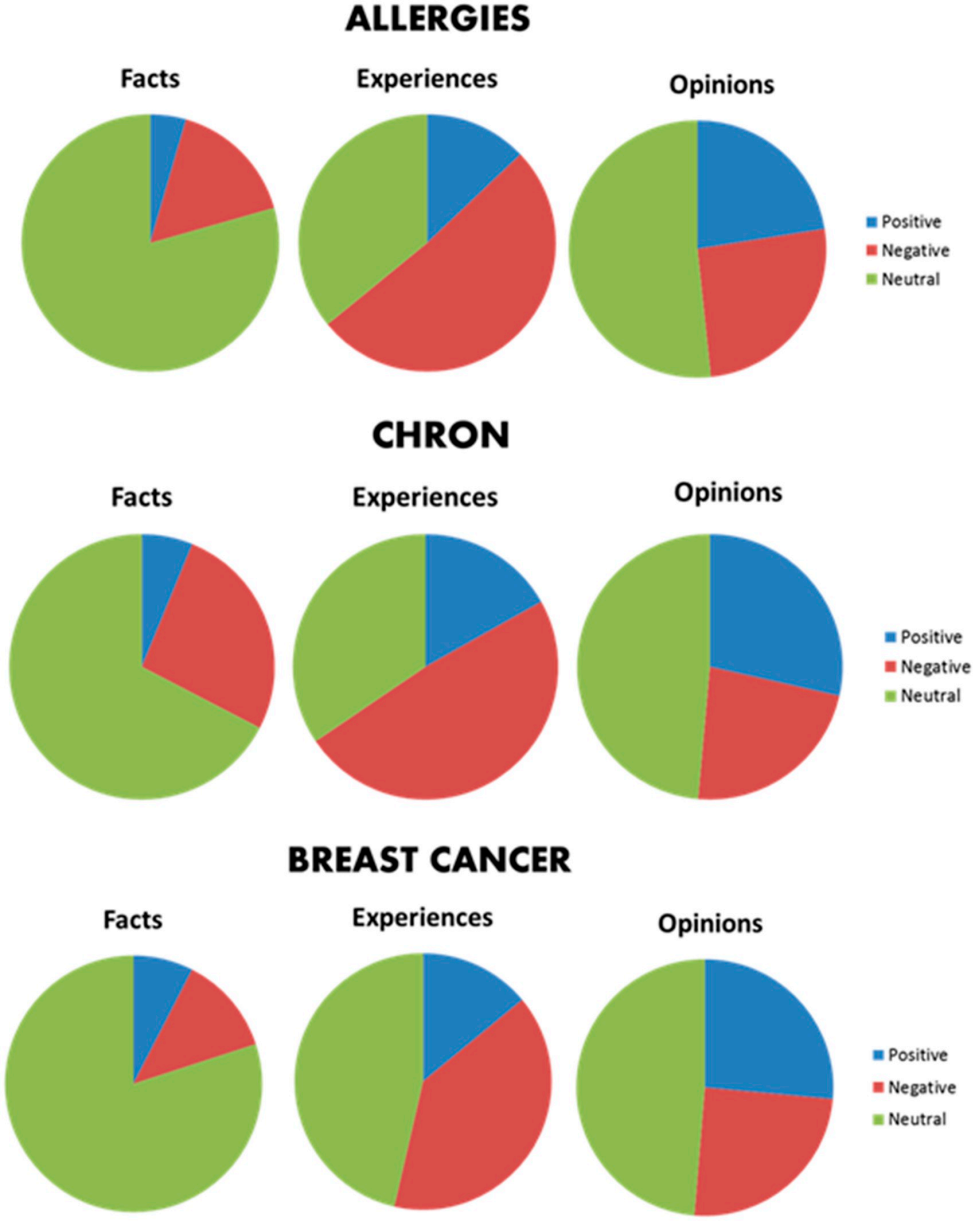
Distribution of sentences into polarity classes (“Positive”, “Negative” and “Neutral”) and factuality classes (“Fact”, “Opinion” and “Experience”).

The charts in Fig 1 show the distribution of “positive”, “negative” and “neutral” instances between the “fact”, “opinion” and “experience” classes for each disease. As expected, facts are mostly neutral but may also be polar: around 25% of facts in all domains are either positive or negative. This is a considerable amount of information that may not be neglected when classifying polarity. In contrast, experiences and opinions are more polarized. In particular, experiences are mostly negative, what was also expected given the working scenario (i.e., the texts are written by patients of chronic diseases).

Tables 4 and 5 show the average inter-annotator agreement per disease for the two groups of labels. We have calculated agreement for each pair of annotators separately, and then computed the average.

**Table 4:** Percent inter-annotator agreement for the three polarity labels and the three diseases.

	Positive	Negative	Neutral
**Allergies**	79%	76%	71%
**Crohn**	68%	68%	79%
**Breast cancer**	77%	70%	79%
**Average**	75%	71%	76%

**Table 5:** Percent inter-annotator agreement for the three factuality labels and the three diseases.

	Experience	Opinion	Fact
**Allergies**	86%	69%	65%
**Crohn**	88%	65%	72%
**Breast cancer**	77%	70%	79%
**Average**	84%	68%	72%

Comparing agreement per label, we see that the highest degree of agreement is reached for the “Experience” label, which corresponds to the majority label for the factuality category. The worst agreement was achieved for the “Opinion” label. Experiences seem to be the easiest to identify: they clearly describe something that has happened to the patient, while the boundary between facts and opinions is, in this scenario, not always clear: sometimes, for instance, a patient presents her opinion on a treatment as a refuted fact, but it is just a personal impression that can not be proved. In contrast, there was no significant differences between the results of the inter-annotator agreement for the three different polarity labels. Moreover, agreement achieved is similar to those reported in other 3-classes text classification problems [15, 60].

### Feature types

In order to represent the patient-authored text, we have extracted and tested features belonging to 6 broad groups or categories of information: content-based features, sentiment-based features, grammatical features, network-based features, domain-specific features and factuality-based features.

#### Content-based features

Content-based features considered include words embeddings, concept embeddings and bag-of words.

- **Word embeddings** (W2V): Word embeddings using Word2vec [39] have been extensively used to measure the semantic similarity between words. Our word embeddings comprise the pretrained vectors published by [61],which have been extensively used in NLP tasks. For each sentence we created an averaged sum of the word vectors—each word in the sentence was used to obtain its word embeddings and we summed over all the word vectors within the sentence. We use an embedding size of 400 dimensions. We ignored punctuations. The entries in the sum vector were used as features.
- **Concept embeddings** (C2V): Concept embeddings are computed following the same procedure as for word embeddings, but instead of using words extracted from the posts, text is first mapped onto concepts from the UMLS Metathesaurus using MetaMap (https://metamap.nlm.nih.gov/). It is worth mentioning that the UMLS Metathesaurus is a compendium of health-related vocabularies, including the so called Consumer health vocabularies (CHVs), which have been developed to aid health informatics applications to deal with patient authored text. This makes the vocabulary specially suitable for our purpose.
- **Bag of words** (BoW): We represent each sentence as a vector where each feature is the TF*IDF value of a term within the dataset. TF*IDF is computed as follows:
  $$w_{i,j}=tf_{i,j}\times log\left(\frac{N}{df_{i}}\right)$$
  where:
  *tf*_*i*,*j*_ is the number of occurrences of *i* in *j*;
  *df*_*i*_ is the number of documents containing *i*;
  and *N* is the total number of documents.

#### Domain-specific features

Domain-specific features are based on the UMLS knowledge base. In particular, we extract UMLS Semantic Types (ST) and represent each sentence using their TF*IDF values of the semantic types. A semantic type is a broad subject category to which the UMLS Metathesaurus concepts are assigned to. Examples of semantic types are “Disease or syndrome”, “Body Location or Region” and “Chemical”.

#### Positional features (Pst)

We have calculated two positional features and tested their joint effect: **the position of the sentence** within the post, and **the position of the post** within the thread.

#### Network features (Net)

The following two network features have been jointly considered:

- The **number of replies of the post**, which is an indication of the popularity of the topic dealt within it.
- **Is a primary question**: a binary feature that is 1 if the sentence belongs to the first post in a conversation, and 0 otherwise.

#### Sentiment-based features (SA)

Two features traditionally used to separate facts from opinions and to determine the polarity of such opinions have been tested: the number of positive/negative words and the emotions expressed in the text.

- **Number of positive/negative words**: number of positive and negative words within the sentence are extracted using three affective lexicons: the General Inquirer [26], SentiSense [27] and SentiStrength [28].
- **Emotions expressed in the text**: the text is represented as the frequency of the different emotions, according to the SentiSense lexicon, that are found within the text. This lexicon includes the following emotions: *Ambiguous*, *Anger*, *Calmness*, *Despair*, *Disgust*, *Anticipation*, *Fear*, *Hate*, *Hope*, *Joy*, *Like*, *Love*, *Sadness*, and *Surprise*. Previous works have shown that using fine grained emotions to represent texts (rather than polar values) allows for significant improvements on polarity classification [23].

#### Grammatical features (Gramm)

Grammatical features are also commonly used as classification features in sentiment analysis tasks. In particular, the part-of-speech of words in the text and the presence of negations have proven to be useful [29, 30, 62]. Moreover, we want to test if the verb tenses within the sentence may help to predict its information type. Grammatical categories of words within a sentence are assigned using the Stanford parser.

- **Verb tense**: Our hypothesis is that past tense verbs are more frequent when expressing an experience, while facts are most frequently expressed using present tense verbs and advices are usually given using imperative forms. We want to test if this feature may indirectly help to predict polarity.
- **Part-of-speech**: The frequency of verbs, nouns, adverbs and adjectives are used as classification features.
- **Negation**: negation is implemented as a binary feature that indicates the presence of a negation in the sentence, and detected using a list of negation tokens from [32].

#### Factuality (Fact)

Factuality represents the manual label assigned to each sentence within the corpus that indicates whether the sentence is a “fact”, an “opinion”, or an “experience”. As we have observed from the distribution of sentences in classes in the eDiseases dataset (see Fig 1), “facts” and “experiences” are, in general, more likely to be neutral than “opinions”. Thus, we want to test if we could improve classification by previously detecting the factuality of the sentence.

### Machine learning algorithms

We have used different learning algorithms implemented in the Weka data-mining software (http://www.cs.waikato.ac.nz/ml/weka/) with the various feature sets described in the previous section.

In particular, we have tested the following algorithms:

- **Sequential Minimal Optimization** (SMO): is an algorithm for implementing Support vector Machines (SVM) that solves the quadratic programming problem that arises during the training process. A SVM is a non-probabilistic, binary linear classifier, that tries to achieve the best separation of data by an hyperplane that is maximally distant from them. It presents a robust performance with respect to sparse and noisy data and has been extensively applied to text classification [63, 64] and sentiment classification [65, 66]. We use the default loss function in Weka for SVM, which is called hinge loss, so that, for an intended output *t* = ±1 and a classifier score *y*, the hinge loss of the prediction *y* is defined as *l*(*y*) = *max*(0, 1 − *t* × *y*). In the case of a multi-class problem, the function is reformulated as *l*(*y*)) = *max*(0, 1 + *max*_*t*≠*y*_*w*_*t*_ × *x* − *w*_*y*_ × *x*).
- **Naive Bayes** (NB): Naive Bayes is a simple probabilistic classifier based on applying Bayes theorem with strong independence assumptions between the features (it assumes that the value of a particular feature is independent of the value of any other feature, given the class variable). It predicts membership probabilities for each class such as the probability that given record or data point belongs to a particular class. The class with the highest probability is considered as the most likely class. This is also known as Maximum A Posteriori (MAP). Despite its simplicity, Naïve Bayes classifiers have worked quite well in many complex classification tasks, including health text classification [67] and sentiment classification [68]. We execute Naive Bayes with the Weka default parameters.
- **Random Forest** (RF): Random Forest is an ensemble classification method. It produces a different classification tree at each iteration from a random subset of the data, and at each node in the tree, a random subset of predictor variables are selected. Multiple trees are constructed in this way. At test time, the classification of these individual trees are combined to get the final prediction. Despite its simplicity, random forest classifiers have been widely applied for sentiment classification [69] and also in text classification [70]. We use the default settings in Weka that produce 100 trees with an unlimited maximum depth.
- **Vote**: Vote is a machine learning meta-algorithm that combines other classifiers using different combinations of probability estimates. Previous works have stated that combining classifiers may lead to better performance [71], even in text classification tasks [72]. We use the Vote algorithm with the SMO, Naive Bayes and Random Forest classifiers and the default parameters in Weka, which means that the output of the different classifiers are combined using the average of their probabilities.

## Evaluation and results

### Experiments

To evaluate the different combinations of features, we use *accuracy* and *F-measure*, as traditionally done in supervised classification. Accuracy and F-measure are defined as follows:

**Accuracy** is the proportion of true results (both true positives and true negatives) among the total number of cases examined, and is computed as follows:

$$Accuracy=\frac{true\;positive+true\;negative}{true\;positive+true\;negative+false\;positive+false\;negative}$$ (1)

**F-measure** is the harmonic mean of precision and recall, and is computed as follows:

$$F-measure=\frac{2\times recall\times precision}{recall+precision}$$ (2)

where **precision** is defined as:

$$precision=\frac{true\;positive}{true\;positive+false\;positive}$$ (3)

and **recall** is:

$$recall=\frac{true\;positive}{true\;positive+false\;negative}$$ (4)

Evaluation is performed using cross-fold validation with Weka, for each of the three diseases in the dataset separately, and for the three diseases together. We use the stratified cross-fold validation technique, with K = 10. This means that the original sample is partitioned in k equal size subsamples, but ensuring that each fold has, approximately, the correct proportion of each of the class values. Of the k subsamples, the first subsample is used for testing the model, and the remaining k-1 subsamples are used as training data. The cross-validation process is then repeated k times (the folds), with each of the k subsamples used exactly once as the test data. The k results from the folds are then averaged to produce a single estimation. This is a very precise technique that reduces the variance of the estimate, especially in the case of small datasets.

We also show the result of predicting the majority class, in order to detect a common problem for learning algorithms that optimize learning for accuracy (they may be simply predicting the majority class).

We calculate which results are statistically significant by applying the Chi-square test with the FDR (false discovery rate) method [73]. This method was developed for controlling type I errors when multiple hypotheses are tested. In short, this method ranks the hypothesis by their P values. Each hypothesis of rank r is compared with a significance cutoff, now called a false discovery rate (FDR), divided by (n-r). In this work, we use FDR of 0.001, 0.01 and 0.05 to determine statistical significance.

### Results

Tables 6–8 show the classification results for the three different diseases: allergies, crohn and breast cancer, respectively. In each table, it is possible to separate three broad groups of experiments, which correspond with the combination of the bags of words (BoW), word embeddings (W2V) and concept embeddings (C2V) features (that we will refer to as *primary features*), with the domain-based (ST), positional (Pst), network-based (Net), sentiment-based (SA), grammatical (Gramm) and factuality features (Fact) (that we will refer to as *secondary features*). We use this distinction to facilitate the analysis of the results. We also compare the results of the different ML algorithms.

**Table 6:** Feature comparison for the allergies domain. Results are reported in Accuracy and F-measure. Best results are indicated in bold. For each group of experiments, significance of the best combination of features with respect to the baseline (BoW, W2V and C2V, respectively) is calculated (FDR<0.001***, FDR<0.01**, FDR<0.05*).

	SMO		RF		NB		Vote	
Feature	Acc	F-1	Acc	F-1	Acc	F-1	Acc	F-1
BOW	56,9	56,3	62,1	57,6	64	62,9	63,9	62,6
BOW + ST	59,6	59,2***	63,5	59,6	64,1	63	63,4	62,1
BOW + ST + Pst	59,6	59,7***	62,9	58,4	64,3	63,1	63,6	62,3
BOW + ST + Pst + Net	59,5	59,7***	61,5	57,2	64,2	63	63,4	62
BOW + ST + Pst + Net + SA	60,1	59,5***	61,8	57,6	65,1	64,2	65	64
BOW + ST + Pst + Net + SA + Gramm	61,2	60,8***	64,4	61,1	65,2	64,4	65,9	64,9
BOW + ST + Pst + Net + SA + Gramm + Fact	64,7	64,8***	62,2	59,6	65	64,1	**66**,**1**	**65**,**2**
W2V	66,2	65	63,5	59,1	53,9	44,2	63,9	61,7
W2V + ST	66,8	66	63,8	59,4	59,9	54,3	64,4	62
W2V + ST + Pst	64,5	63,6	61,1	57,1	58,1	53,4	63,4	61
W2V + ST + Pst + Net	64,6	63,7	62,9	59	57,9	53,3	63,7	61,2
W2V + ST + Pst + Net + SA	67	66,2	63,2	59,5	59,3	55,6	64,6	62,6
W2V + ST + Pst + Net + SA + Gramm	66,6	65,8	61,8	57,3	58,6	55,4	64,6	62,6
W2V + ST + Pst + Net + SA + Gramm + Fact	**68**,**1**	**66**,**2**	63,1	59,4	61,8	59,6	65	63,3
C2V	62,3	60,8	61,7	57,3	54,3	45,3	61,5	58,5
C2V + ST	65,9	65,2**	61,8	57,4	57,9	53,3	64,1	61,7
C2V + ST + Pst	66,2	65,6**	61,2	57,1	58,1	53,4	63,4	61
C2V + ST + Pst + Net	66,1	65,4**	62,9	59	57,9	53,3	63,7	61,2
C2V + ST + Pst + Net + SA	**66**,**6**	**66**,2**	63,2	59,5	59,3	55,6	64,6	62,6
C2V + ST + Pst + Net + SA + Gramm	62,4	61,6	61	56,1	59,2	56,2	62,8	60,8
C2V + ST + Pst + Net + SA + Gramm + Fact	63,2	62,4	63,4	59,4	61,8	59,8	63,4	61,5

**Table 7:** Feature comparison for the crohn domain. Results are reported in Accuracy and F-measure. Best results are indicated in bold. For each group of experiments, significance of the best combination of features with respect to the baseline (BoW, W2V and C2V, respectively) is calculated (FDR<0.001***, FDR<0.01**, FDR<0.05*).

	SMO		RF		NB		Vote	
Feature	Acc	F-1	Acc	F-1	Acc	F-1	Acc	F-1
BOW	57,9	56,8	61,7	60,7	63,1	62,4	64	63,3
BOW + ST	61,2	59,8	62	60,8	63,1	62,3	64,5	63,7
BOW + ST + Pst	61,2	59,8	63,1	61,5	62,8	62	64,1	63,3
BOW + ST + Pst + Net	61,2	59,8	62,7	61	63	62,2	64,1	63,3
BOW + ST + Pst + Net + SA	63,8	62,8	64,3	61,8	64	63,1	64,4	63,6
BOW + ST + Pst + Net + SA + Gramm	64,4	63,4*	63,6	61,9	64,6	63,7	65,4	64,5
BOW + ST + Pst + Net + SA + Gramm + Fact	**65**,**5**	**64**,6**	64,6	62,8	63,8	62,7	66	65,2
W2V	65,8	65,4	61,9	59,3	52,2	46	64,4	62,9
W2V + ST	66,2	65,9	62	59,4	54,8	50,2	64,9	63,4
W2V + ST + Pst	65,7	65,4	63,3	60,5	55	50,5	64,7	63,4
W2V + ST + Pst + Net	65,2	64,9	61,8	59,2	55	50,5	66	64,7
W2V + ST + Pst + Net + SA	67	66,3	61,8	59,2	56,1	56,1	66,2	65,2
W2V + ST + Pst + Net + SA + Gramm	66,4	66,3	63,1	60,3	59	59	65,8	64,5
W2V + ST + Pst + Net + SA + Gramm + Fact	**67**,**2**	**66**,**4**	62,8	60,3	56,9	56,9	65,2	64,1
C2V	61,2	60,6	59,1	56,9	50,3	50,3	60,5	58,8
C2V + ST	65,2	64,9***	62	59,4	54,8	54,8	64,9	63,4
C2V + ST + Pst	65,7	65,4***	63,3	60,5	55	55	64,7	63,4
C2V + ST + Pst + Net	65,2	64,9***	61,8	59,2	55	55	65,9	64,7
C2V + ST + Pst + Net + SA	**66**,**8**	**66**,5***	61,8	59,2	56,1	56,1	66,2	65,2
C2V + ST + Pst + Net + SA + Gramm	62,9	62,5**	60,1	57,5	55,9	55,9	62,7	61,4
C2V + ST + Pst + Net + SA + Gramm + Fact	62,9	62,6**	60,1	57,7	58,1	58,1	64	62,7

**Table 8:** Feature comparison for the breast cancer domain. Results are reported in Accuracy, F-measure, Precision and Recall. Best results are indicated in bold. For each group of experiments, significance of the best combination of features with respect to the baseline (BoW, W2V and C2V, respectively) is calculated (FDR<0.001***, FDR<0.01**, FDR<0.05*).

	SMO		RF		NB		Vote	
Feature	Acc	F-1	Acc	F-1	Acc	F-1	Acc	F-1
BOW	57,1	47,7	57,6	46,1	58,1	49,4	58,4	48,9
BOW + ST	60,9	60,1***	64,4	59,4	63,9	62,9	63,4	61,5
BOW + ST + Pst	61,8	59,7***	64,8	59,9	63,6	62,6	63,9	62,4
BOW + ST + Pst + Net	59,8	59,3***	64,7	59,9	63,5	62,7	62,9	61,3
BOW + ST + Pst + Net + SA	62,1	61,6***	64,2	59	65,3	64,5	**65**,**5**	**64**,**1**
BOW + ST + Pst + Net + SA + Gramm	56,6	47,1	57,2	55,5	57,6	49,2	59,3	52,4
BOW + ST + Pst + Net + SA + Gramm + Fact	57,1	49,2	56,7	55,3	57,5	49,7	59,7	53,6
W2V	65,9	65,1	62,8	56,5	57,5	46,4	66,5	63,5
W2V + ST	66,2	65,2	63,1	56,4	60,3	62,9	67	63,4
W2V + ST + Pst	66,5	65,9	61,3	53,8	60,6	62,6	67,3	63
W2V + ST + Pst + Net	66,1	65,4	62,3	55,2	59,9	62,7	67,4	63,4
W2V + ST + Pst + Net + SA	68,4	66,5	62,6	55,7	61,9	64,5	**68**,**7**	**65**,**3**
W2V + ST + Pst + Net + SA + Gramm	66	65,8	62,9	56,5	62,6	49,2	68,2	65,9
W2V + ST + Pst + Net + SA + Gramm + Fact	64,7	64,5	63,3	56,9	62,6	49,7	68,5	66,4
C2V	62,6	60,8	61,2	55	57,4	45,6	63,8	59,6
C2V + ST	65	65,2**	63,1	56,4	63,9	53,8	65	61,4
C2V + ST + Pst	66,5	65,9**	61,3	53,8	63,7	54,4	65,3	62
C2V + ST + Pst + Net	66,1	65,4**	62,3	55,2	63,5	54,1	66,4	63,4
C2V + ST + Pst + Net + SA	**67**,**8**	**67**,2**	62,6	55,7	65,3	57,6	67,7	65,3
C2V + ST + Pst + Net + SA + Gramm	62,4	61,6*	63,1	56,5	57,6	58,8	64,8	62
C2V + ST + Pst + Net + SA + Gramm + Fact	63,2	62,4*	63,3	57	57,5	59,5	65,3	62,5

#### Comparison of machine learning algorithms

Concerning the ML algorithms, our experiments show that, overall, SMO and Vote perform much better than Naive Bayes, and slightly better than Random Forest. Vote, however, usually improves the performance over SMO but sometimes this is achieved by penalizing recall. SMO, in contrast, produces very balanced precision and recall. In our particular scenario, there is not a preference for optimizing precision or recall. It is interesting that NB produces results similar to those of SMO and Vote when the BoW features are used, but produces very poor results when embeddings are used as attribute.

#### Comparison of primary features

Concerning the primary features (BoW, W2V and C2V), Tables 6–8 show that the best results are obtained when **word embeddings** are used; followed by the **concept embeddings** and, finally, by the **bags of words**. Results for W2V and C2V are significantly better (p <0.05) than those for BoW. This is true for all the three diseases. It is interesting that the use of concept embeddings produces lower results than the use of word embeddings, even though UMLS concepts are expected to more accurately represent the domain knowledge and help to deal with semantic issues such as synonymy and lexical ambiguity [74]. The main reason for this finding is that, as stated in [75], an important proportion of the vocabulary used by patients are not found in terminologies and ontologies. This has been empirically checked during our experimentation (despite the use of the consumer health vocabulary). Another reason is that the texts present frequent typos and orthographic errors.

#### Comparison of secondary features

We next combine each of the primary features above (BoW, W2V and C2V) with the secondary features (ST, Pst, Net, SA, Gramm and Fact) and check if these secondary features may help to improve the performance of the primary ones. We analyze the effect of each individual feature below:

The use of the **UMLS semantic types** (ST) as classification features has a positive impact in performance. Semantic types classify the vocabulary in categories, such as sign or symptom, disease or syndrome, dysfunctions, laboratory procedures, injury or poisoning, etc., that provide useful information for polarity classification: e.g., having an injury is usually negative, a laboratory procedure may be positive or negative depending on the result (but barely neutral), etc. However, the impact is still lower than expected, especially for the W2V representation. We find several reasons for this: first, since we are dealing with non-expert generated contents, it is expected that a great deal of the vocabulary will not be domain-specific. This kind of vocabulary is not likely to be mapped to semantic types in the UMLS Semantic Network, and as a result, a lot of potentially important information is missed; second, as already mentioned, typos and orthographic errors are very frequent in patient-generated texts; and third, representing the text as a set of semantic types means representing medical concepts at a very high level of generalization that usually leads to a decrease in precision (and so F-measure). Similar results were found by [76].

When the **positional features** (Pst) (the position of sentences within the post and the position of the post within the thread) are combined with BOW, W2V and C2V features, respectively, performance remains unchanged or the change (positive or negative) is not significant. It seems, therefore, that the position of the sentence in the post is not a good estimator of its polarity. The same results and conclusions are obtained in the case of the **network-based features** (Net) (number of replies of the post and “is a primary question”): according to our experiments, it is not expected for more negative or positive posts to get a more active participation or impact in the community, although our initial intuition was that the more negative a post was, the more support or relevance it gets.

In contrast, results in Tables 6, 7 and 8 show that, for every disease, **sentiment-based features** (SA) are the most influential. This was expected, since the task is to classify text into different polarity classes. It is worth remembering that sentiment-based features include both the number of positive/negative words that are found within the text and the emotions that are expressed within it. These features have already been shown to be of great use to perform polarity classification, both in general and health specific text. However, we have observed that a good number of words (especially those that are domain-specific) cannot be mapped to any emotion/polarity value in the lexicons (which are general purpose). We hypothesize (and this will be part of our future work) that using a domain specific polar lexicon would help to improve the classification results.

Concerning the **grammatical features** (Gramm), which include the form of the verbs within the sentence (present/ past/ imperative), the part of speech of the words within it, and the presence of negations, our experiments show that they do not help to predict polarity and even produce a decrease in performance. On the one hand, the form of the verb and the part of speech of words, which have shown to be very helpful for discerning between factual and opinionated information [77] do not seen to be useful for predicting polarity, even though, in our dataset, facts and opinions have an uneven distribution of polar classes. On the other hand, to evaluate the real effect of negation, a more sophisticated mechanism of detection should be employed, so that, at least, polarity of words within the scope of such negation is changed. Only detecting the presence of negation tokens does not produce any improvement.

Finally, the effect of the **factuality** (Fact) feature is quite homogeneous across domains and combinations of features: it increases performance for nearly all feature combinations and diseases, but the improvement is not significant. This was expected, given that a correlation exists between polarity and factuality classes in the dataset (see Fig 1). However, it should be noted that this factuality label has been manually assigned, which is a limitation for a practical application.

It is also worth noting that the effect of adding new features is more marked for the BoW feature than for the W2V and C2V features, that is, for the feature that individually produces poorer classification results. Since the BoW model does not capture the semantics properly, it benefits from the use of other features.

#### Comparison among different diseases

In order to compare the performance between different diseases, Table 9 shows a summary of the results in previous tables. The results for the three diseases are very similar, but slightly higher for breast cancer and allergies than for chron. For all the three diseases, the best results are obtained by the word embeddings approach (in combination with other sets of features).

**Table 9:** Comparison between diseases (allergies, crohn, and breast cancer) (Average accuracy) for the SMO classifier.

Feature	Allergies	Crohn	Breast cancer
BoW	56,9	57,9	57,1
W2V	66,2	65,8	65,9
C2V	62,3	61,2	62,6
Best combination of features	68,1	67,2	68,4
Majority baseline	53,8	43,6	56,7

These results are slightly lower than those obtained in other sentiment analysis tasks, such as polarity classification of online product reviews, where accuracy is around 75%. This is due to (i) the difficulties that the domain entails and that have already been mentioned, and (ii) the highly unbalanced classes in the dataset. Similar results were found in other domain-specific sentiment analysis tasks, such as reputation polarity classification, where classes are also very unbalanced [15].

In order to see what is the real effect of having imbalanced classes, we have made a further experiment. We have balanced the classes using the “Re-sample” Weka filter (under-sampling). Table 10 shows the results and proves that equilibrating the number of instances per class has a very positive impact in the classification performance, allowing for (a) averaged accuracies around 70%-80%, and (b) similar performance for the three individual classes.

**Table 10:** Average F-1 by class for the SMO-W2V classifier (Resample). Best results are indicated in bold.

Class	Allergies	Crohn	Breast cancer
Neutral	**82**,**6**	72,5	74,6
Positive	71,8	**79**,**2**	**81**,**4**
Negative	74,9	72,18	80,9
Average	76,6	75,1	79,0

When balancing the number of instances per class, we also see that the classification results become similar for the three diseases. Still, the best average F-1 is obtained for breast cancer, next allergies, and finally chron. F-1 per class is well-balanced for the three classes.

#### Combining the different diseases

So far we have considered each disease in isolation. In the following experiment we study how combining the data from all the three diseases affects the classification. In this way, we aim to understand how adaptable the classifiers are to previously unseen diseases. Table 11 shows the results for the best combinations of features.

**Table 11:** Classification results when data for the three diseases are combined for the SMO. Best results are indicated in bold.

Feature	Acc	F-1
W2V	67,2	66,3
W2V + ST + Pst + Net + SA + Gramm + Fact	67,3	66,7
W2V - *Resample*	70,9	71,0
W2V + ST + Pst + Net + SA + Gramm + Fact - *Resample*	73,1	73,2
C2V	64,2	62,8
C2V + ST + Pst + Net + SA + Gramm + Fact	64,9	64,2
C2V - *Resample*	67,9	67,9
C2V + ST + Pst + Net + SA + Gramm + Fact - *Resample*	70,7	70,8

It can be observed in Table 11 that classification results are equivalent to those obtained for the individual diseases. These results suggest that it is possible to apply the classifiers to new diseases (where the distribution of data is expected to be different) and still obtain a good performance. This means that the features learned are robust to variations across domains.

#### Separating facts, experiences and opinions

We have performed a further experiment where we evaluate the classification of polarity when the different types of sentences (facts, opinions and experiences) are considered separately. The results of this experiment are shown in Table 12 and demonstrate that not only opinionated information has a polar orientation, but also factual information. Moreover, in the light of these results, it seems that it is easier to detect the polarity of objective information than that of subjective information. This way, the best F-measure is obtained for the factual sentences, followed by the sentences describing experiences and, finally, by the opinionated sentences. During our experimentation, we have observed that, in our scenario, facts and experiences usually describe the presence and evolution of sign or symptoms, the prescriptions and results of tests and treatments, etc., which usually conveys positive or negative connotations that are easier to identify that those expressed in opinions (which are more personal and subjective).

**Table 12:** F-1 by factuality class and disease for the W2V classifier. Best results are indicated in bold.

Disease	Facts	Experiences	Opinions
**Allergies**	**81**,**3**	79,7	62,0
**crohn**	**80**,**0**	76,1	78,1
**Breast cancer**	**76**,**0**	74,5	67,5

#### Cost sensitive evaluation

Finally, we repeat the experiments in Table 10 in a cost-sensitive evaluation setting. To this end, we use the cost-sensitive evaluation facility in Weka, and establish the cost of misclassifying the class POSITIVE to the class NEGATIVE (and vice versa) to 2, being 1 the cost of misclassifying instances from a polar class to the NEUTRAL class. With these experiment, we want to recognize that the costs caused by different kinds of errors are not assumed to be equal. Results are shown in Table 13. As expected, F-measure decreases for the neutral class, but increases for the negative and positive classes. When the confusion matrix is analyzed, we observe a significant drop in the number of positive instances that are classified as negative (and vice versa).

**Table 13:** Average F-1 by class for the SMO-W2V classifier (*Resample*). Best results are indicated in bold.

Class	Allergies	crohn	Breast cancer
Neutral	72,7	71,6	73,2
Positive	**82**,**4**	**76**,**9**	79,7
Negative	77,3	71,7	**80**,**6**
Total	77,5	73,4	77,8

## Discussion

The extensive experimentation performed shows that it is possible to efficiently classify patient-authored contents according to the sentiments they convey, achieving an accuracy around 70% for the three-classes categorization of polarity. These results are, however, slightly lower than those achieved in other domains, such as polarity prediction in products and service reviews. Further *ad-hoc* experimentation and analysis is needed to improve classification of patient-authored information.

The automatic categorization of patient-authored texts has interesting practical applications. It may be used, for instance, in combination with information extraction techniques to detect positive experiences with a medication, to identify negative opinions on different treatments or procedures, or to know about negative facts associated to a given symptom. In this way, users can benefit of quicker access to the issues they are concerned about.

Using the traditional feature of bags of words provides a very strong baseline, although word and concept embeddings allow for significantly better results. In the case of the C2V representation, however, results are lower than expected. This is due to the fact that the language used by patients when sharing their opinions and experiences in social networks is not specialized medical language, but a mix of colloquial speech and medical terminology. While specialized medical terms are expected to be found in the UMLS metathesaurus, other non-specialized terms are not expected to be mapped to any concept. Moreover, some of such terms may be of great interest to the classification of polarity (e.g., adjectives and adverbs).

However, the best performance is achieved by the W2V approach for the three different diseases. The word embeddings model extends the bag of words model by incorporating context, and provides significant improvements in classification performance. In addition, adding other features to the bags of words, concept embeddings and word embeddings representations improves performance in a very small percentage. However, not all features contribute equal: the SA (sentiment analysis features) and the ST (semantic types) usually allow for significant improvements. The best performance are the SA features, which include the number of positive and negative words and the emotions expressed in the text according to different general-domain affective lexicons. This result, even though expected, is interesting since it suggests that using a domain-specific affective lexicon may lead to better classification results. The Factuality feature produces non-significant improvements, while Grammatical, Network-based and Positional features do not have any impact on classification. Grammatical features even affect negatively the classification performance.

We have seen that the classes are very unbalanced. It seems that, when patients share information in online communities, most of the information is neutral (i.e. it has no positive or negative connotations). The imbalanced dataset problem is quite common in many real applications, and may lead to poor performance for the machine learning algorithms, since they tend to be biased towards the majority class [78]. To understand the extent of this problem, we have applied an under-sampling strategy that consists in randomly removing examples from the majority class to make the dataset balanced. When applying this re-sampling strategy, classification accuracy considerably increases for the three diseases (an increment of around 10 percentage points of accuracy) (see Table 10).

On the other hand, our experiments suggest that it is possible to apply the classifiers to previously unseen diseases, without carrying out any adaptation process. This means that the features learned show good cross-domain generalization performance.

Finally, we have empirically checked that not only opinionated information has a polar orientation but also factual information. In particular, we found that around 25% of the information that patients share in health-related social networks are facts, and that around 50% of these facts are either positive or negative (in terms of the sentiments and emotions that they evoke in the patients). We also found that it is easier to detect the polarity of objective information than that of subjective information.

## Conclusions

Due to the recent development of online health communities and forums, research in information extraction and classification in these media is becoming increasingly popular. Surveys show that patients and caregivers significantly benefit from social interaction with peers and from the sharing of knowledge, experiences and support [79], but the challenge is to make the huge amount of information available accessible and useful.

In order to facilitate the access to this valuable information, in this work we evaluate the feasibility of exploiting words embeddings together with lexical, syntactic, semantic, network-based and emotional properties of texts to classify patients-generated contents according to their polarity (i.e., positive, negative, neutral). Unlike previous works from the sentiment analysis field that consider that only subjective information may have polar orientation, we hypothesize that, especially when dealing with medical information, objective or factual statements have also polar connotations and this information is particularly important for users. In this way, for instance, patients looking for information about a drug are not only interested in the negative opinions and experiences of other patients, but also in the contrasted facts about the side effects of such drug. These contrasted side effects are clearly negative from the perspective of the patient that is taking the drug.

To perform our experiments, we have annotated a set of more than 3500 sentences from online health forums of breast cancer, crohn and different allergies, respectively. Each sentence in a post is manually labeled as “experience”, “fact” or “opinion”, and as “positive”, “negative” and “neutral”. This dataset will be made available for the research community.

Our experiments suggest two important results: first, it is possible to predict polarity of patient-authored sentences with a very high accuracy (around 70 percent) using a combination of lexical features, sentiment-based features and word embeddings; and second, when dealing with medical information, negative and positive facts (i.e. objective information) are nearly as frequent as negative and positive opinions and experiences (i.e. subjective information), and polarity of such facts may be predicted more accurately that that of opinions and experiences.

As future work we plan to build a domain-specific emotional lexicon that maps domain words to their polarity and/or sentiment. This lexicon must also include domain-specific polar facts. We expect that such a lexicon will allow us to improve the performance of the sentiment-based features for classification. We will also test new features such as emoticons, which have shown to be very useful in the identification of emotions and sentiments [80]. As a long-term future work, our aim is to go a step further in facilitating patients’ access to relevant information by building a system that allows for the automatic identification of drugs and treatments in patient conversations, so that opinion, facts and experiences of patients on such treatments may be detected and automatically classified into positive and negative. Another interesting line of future work will be the automatic detection of adverse events for a drug mention in a social media post. We also plan to introduce dialog structure analysis to address the problem at the conversational level.
